# Prevalence and Association of Malaria With the Blood Group on Febrile Patients at Woldia Comprehensive Specialized Hospital, Northeast Ethiopia

**DOI:** 10.1155/2024/9942758

**Published:** 2024-09-28

**Authors:** Wagaw Abebe, Fasikaw Wudu, Gebreeyesus Derib, Foziaya Fentie, Agenagnew Ashagre

**Affiliations:** Department of Medical Laboratory Sciences College of Health Sciences Woldia University, Woldia, Ethiopia

**Keywords:** ABO blood group, Ethiopia, malaria, prevalence, Woldia

## Abstract

**Background:** Malaria is a disease transmitted by vectors and caused by unicellular *Plasmodium* parasites. Malaria pathogenesis is associated with the ABO phenotype. However, there is little information on the frequency of malaria disease and its relationship with the ABO blood group in the study area. Therefore, the purpose of this study was to determine the prevalence of malaria infection and its association with the ABO blood group at Woldia Comprehensive Specialized Hospital.

**Method:** An institutional-based cross-sectional study was conducted from December 3, 2022, to February 30, 2023. Convenient sampling was used for selecting the study participants. To identify malaria parasites, thick and thin blood films were made. Additionally, blood was drawn to identify the ABO blood group type. Before being analyzed with SPSS software Version 27, the data was coded and entered into EpiData Version 3.1. To ascertain the variable's association, a logistic regression was done.

**Results:** Out of 192 patients that attended Woldia Comprehensive Specialized Hospital, 16 (8.3%) were found to be infected with *Plasmodium* parasites using microscopy. Among them, 9 (4.7%), 5 (2.6%), and 2 (1.0%) had *Plasmodium falciparum*, *Plasmodium vivax*, or mixed infections, respectively. As a result, 30.7%, 25.5%, 24.5%, and 19.3% of the participants had blood types A, B, AB, and O, respectively (AOR = 2.359, 95% CI: 1.03–12.289, *p* = 0.03).

**Conclusion and Recommendation:** The total number of microscopically confirmed malaria parasites was 8.3%. *P. falciparum* was dominant over *P. vivax*. Individuals with blood group O were less likely to get severe malaria than those with other blood groups. Based on the findings of this study, we recommend that additional studies investigate the probable relationship between the ABO blood group phenotype and malaria infection.

## 1. Introduction

Malaria is a vector-borne illness caused by a unicellular parasite from the *Plasmodium* genus. Human malaria is caused by five *Plasmodium* species: *Plasmodium falciparum* (*P. falciparum*), *Plasmodium vivax* (*P. vivax*), *Plasmodium malariae* (*P. malariae*), *Plasmodium ovale* (*P. ovale*), and *Plasmodium knowlesi* (*P. knowlesi*) [1, 2]. According to the data from the World Health Organization (WHO, 2022), there were an anticipated 247 million cases of malaria and 619,000 malaria-related deaths worldwide in 2021. Approximately 95% of malaria cases globally and 96% of malaria-related deaths occurred in the WHO African Region, which reported an estimated 234 million cases of malaria [3]. Nearly 68% of Ethiopia's population, or about 54 million people, resides in areas where malaria poses a threat, and over 75% of the country's geographical regions are classified as malarial [3, 4]. There were 263,476 reported cases of malaria in the southern region of Ethiopia [5]. Malaria cases are of particular concern in the western lowlands of Oromia, Amhara, Tigray, and nearly all of Gambella Regional State, as well as in Benishangul-Gumuz Regional State [6]. The prevalence of malaria in the Amhara Region was 21.9%. Approximately 80% of the region's land is classified as malarial, and data from 1995 to 2020 indicate that 68% of the population is at risk of contracting malaria. Additionally, 27% of Woldia's land area is malarial [7, 8].

Malaria is transmitted through the bite of an infected female *Anopheles* mosquito, which introduces approximately 3000 sporozoites into the person with each feeding [9, 10]. These sporozoites travel to the liver, where many developments take place [11]. Sporozoites enter the bloodstream, travel to the liver, and infect liver cells, where they develop into schizonts. When the schizonts rupture, they release thousands of merozoites into the bloodstream. These merozoites then infect red blood cells (RBCs), and some parasites transform into male and female gametocytes. Infected mosquitoes can then transmit the parasites, perpetuating the cycle [12].

Malaria is a major cause of morbidity and mortality, particularly among children under five and pregnant women [13]. Malaria can lead to anemia and low birth weight due to the loss of prior immunity. It is responsible for approximately 6.5% of abortions, 15% of premature deliveries, and 0.7% of in utero deaths [14]. The symptoms of malaria in humans arise from the invasion and destruction of RBCs by the parasites, as well as the host's immune response. If left untreated, tropical malaria can lead to complications such as cerebral malaria, hypoglycemia, adult respiratory distress syndrome, and severe hemolytic anemia [15].

In addition to its health impact, malaria imposes a significant economic burden worldwide. Total funding for malaria control and elimination was estimated at $3.3 billion in 2020, with contributions from the governments of endemic countries amounting to $1.1 billion, which represents 33% of the total funding (WHO, 2022) [16].

The ABO blood grouping system includes the A, B, and H carbohydrate antigens, along with antibodies against these antigens, while the Rh system is defined by the presence of the D antigen [17]. Consequently, Rh blood grouping is determined by the presence or absence of the D antigen on the surface of RBCs, while ABO blood grouping is based on the presence or absence of the A and B antigens [18, 19].

The ABO blood type system is genetically determined, and the proportions of the various ABO groups vary greatly between populations and ethnic groupings. Blood group antigens are polymorphic features acquired by individuals and groups [20]. Differences in blood group antigen expression can make the host more or less susceptible to a variety of diseases. Blood types can directly influence infection by acting as receptors and/or coreceptors. Polymorphisms in blood types can alter the innate immune response to infection. Several different phenotypes linked with improved host resistance to malaria have been identified in populations living in malaria-endemic areas, as a result of evolutionary forces [21].

The associations between ABO phenotypes and malaria are linked to the disease's pathogenesis, and a deeper understanding of these interactions is essential for exploring the glycobiology of malaria. Typically, individuals with a high parasite density in their peripheral blood are at greater risk of developing severe malaria. However, some individuals can experience severe or even critical malaria with very low peripheral parasitemia due to the sequestration of parasites in deep tissue capillaries [22]. The study has shown that other RBC polymorphisms, such as thalassemia, the absence of erythrocyte complement receptors, glucose-6-phosphate dehydrogenase deficiency, and hemoglobin abnormalities like hemoglobin S and hemoglobin C, can influence susceptibility to and severity of *P. falciparum* malaria [23, 24].

Numerous research studies have been conducted to determine how an individual's ABO blood type influences distinct kinds of malaria. However, contradictory outcomes were discovered [25, 26]. Some research indicated the lack of a substantial connection between malaria and ABO antigens [25, 27]. However, several research found that malaria is more frequent in those with blood groups A, B, and AB compared to those with blood group O [28]. Conversely, other research and reports revealed that persons with blood type O are more vulnerable to malaria infection in endemic locations [29]. However, research on the prevalence of malaria infection and its relationship with the ABO blood group remains limited. Investigating this association is crucial for addressing various issues related to malaria infection and ABO blood group interactions. The ABO blood group's influence on malaria disease's clinical manifestations can aid in the planning of malaria management treatments, in addition to aiding in the understanding of the disease's pathogenesis and clinical morbidity. It can help to establish preventative and control strategies to identify blood types that are more vulnerable to malaria. One possible solution to this problem may be to give priority to treatments for these populations, including bed nets sprayed with insecticide or antimalarial medication. Furthermore, people may make well-informed judgments about their travel and health plans by knowing the connection between blood groupings and malaria risk. For example, those with high-risk blood types should take extra care while traveling to regions where malaria is common. Additionally, studies on the connection between the blood group and malaria may result in the creation of fresh interventions, including vaccinations or therapies tailored to a certain blood group. This might significantly improve the fight against malaria and reduce the number of cases. Thus, the aim of the current study was to evaluate the frequency of malaria infection and how it relates to the ABO blood type.

## 2. Methods and Materials

### 2.1. Study Area

**Figure 1:**
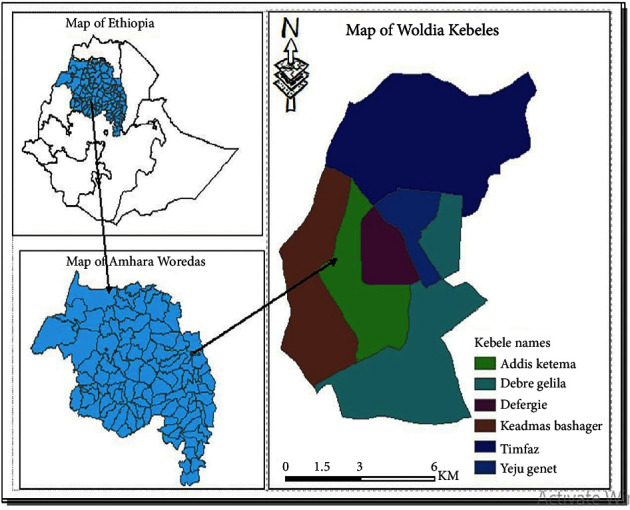
Location map of Woldia [31].

The study was conducted at Woldia Comprehensive Specialized Hospital from December 3, 2022, to February 30, 2023. Woldia is the capital city of the North Wollo Zone, which is located about 360 km from Bahir Dar, the capital city of Amhara Regional State, and 522 km from Addis Ababa, the capital city of Ethiopia. The town has an elevation of 2112 m above sea level. The region has two distinct rainy seasons: the long rainy season, known locally as *kiremt*, which is from June to September, and the short rainy season, known locally as *belg*, which is from February to May. The average annual rainfall in the town is between 525.8 and 919.6 mm, while the seasonal rainfall falls between 256.5 and 701.2 mm and 48.5–255.6 mm during the main growing seasons, June to September and February to May, respectively. The mean annual maximum, minimum, and average temperatures over the study area are 24.7°C, 11.4°C, and 18.4°C, respectively. There were 46,139 people living in Wolida Town overall, with 23,000 men and 23,139 women in 2007 [30]. The town is administratively structured by 10 kebeles, and according to the Ethiopian national census of 2019, the population size of the town is 180,000. Woldia has one comprehensive specialized hospital, two health centers, four health posts, and more than nine private clinics (Figure 1).

### 2.2. Study Design, Period, and Population

An institutional-based cross-sectional study was conducted at Woldia Comprehensive Specialized Hospital from December 3, 2022, to February 30, 2023. The study populations were malaria-suspected participants. Participants who were febrile and suspected to have malaria and those who volunteered to give blood specimens for malaria diagnosis and ABO blood group were included as study participants. The study excluded patients who had received antimalarial drug treatment for the previous 2 weeks and seriously ill patients.

### 2.3. Sample Size and Sampling Technique

#### 2.3.1. Sample Size Determination

The sample size was determined using the single population proportion formula. We used a prevalence rate of *p* = 0.1463 (14.63%) from a study conducted on the prevalence and association of malaria with ABO blood groups in patients at Mekaneeyesus Primary Hospital [32]. A random error of 5% and a confidence level of 95% are assumed in the sample size determination. The sample size was determined by using this formula:

 $$\begin{array}{c} n=\frac{z^{2}pq}{d^{2}} \end{array}$$

where *n* is the sample size; *z* is the confidence level which is 95% (1.96); *p* is an estimate of the prevalence rate of the population, which is 0.1463 (14.63%); *q* = 1 − *p* = 0.8537; and *d* is the margin of error (5% = 0.05).

 $$\begin{array}{c} n=\frac{{\left(1.96\right)}^{2}0.1463\times 0.8537}{{\left(0.05\right)}^{2}} \end{array}$$

 $$\begin{array}{c} n=\frac{3.8416\times 0.12489631}{0.0025}=191.92\sim 192 \end{array}$$

Therefore, the sample size is *n* = 192.

#### 2.3.2. Sampling Technique

The convenience sampling technique was used to select the study participants.

### 2.4. Variables

#### 2.4.1. Dependent Variables

• Prevalence of malaria infection
• Association of malaria infection with ABO blood groups

#### 2.4.2. Independent Variables

• Age
• Gender
• ABO blood group
• Residence
• Stagnant water
• Education
• Bed net

### 2.5. Operational Definition

#### 2.5.1. Febrile Patient

A patient who is febrile is one who has a fever, which is a transient elevation in body temperature above normal.

#### 2.5.2. Malaria-Suspected Patient

An individual exhibiting symptoms suggestive of malaria, such as fever, chills, sweats, headache, muscle pains, lethargy, nausea, vomiting, and diarrhea, is considered a malaria-suspected patient.

#### 2.5.3. Severe Malaria Patient

A patient with severe malaria is defined as having a potentially lethal strain of the parasite together with hypoglycemia (glucose less than 40 mg/dL), hemoglobinuria, respiratory distress, and impaired consciousness or seizures.

### 2.6. Data Collection Procedures and Laboratory Methods

#### 2.6.1. Data Collections

A structured questionnaire was employed to gather data on sociodemographic information and other relevant details. Data collection was conducted through face-to-face interviews with patients and the guardians of children, utilizing closed-ended questionnaires.

#### 2.6.2. Sample Collection and Sample Processing

Capillary blood was collected by finger pricking using a sterile disposable lancet. Heel punctures are used for infants. Immediately, a thin film was spread on the grease-free, frosted-end, labeled slide using a smooth-edged slide spreader. A thick film was also prepared on the same slide and allowed to dry. The thin film was then fixed with methanol. The blood film was stained with 10% Giemsa for 10 min. Then, the blood film was examined by a laboratory technologist using an oil immersion microscope objective (100×), and the result was noted on the data collection form [33].

ABO blood groups were typed by agglutination using commercial antisera (direct method). Two drops of whole blood were placed in different places on a grease-free, clean glass slide, on which a few drops of antisera for blood groups A and B were applied. The blood and the antisera were mixed with a separate applicator stick. The slide will then tilt to detect agglutination, and the result will be recorded accordingly. If agglutination is observed when an individual's blood is mixed with an anti-A reagents, then the individual is said to have blood group “A.” If agglutination is observed when an individual's blood is mixed with an anti-B reagents, then the individual is said to have blood group “B.” If agglutination is observed when an individual's blood is mixed with an anti-A and an anti-B reagent, then the individual is said to have blood group “AB.” If no agglutination is observed when an individual's blood is mixed with anti-A and anti-B reagents, then the individual is said to have blood group “O” [34].

### 2.7. Data Analysis

The data were analyzed using the Statistical Package for the Social Sciences (SPSS) Version 27. A logistic regression test was used to determine the association between ABO blood groups and malaria infection at a 95% confidence interval (CI). Values were considered statistically significant when the *p* value was less than 0.05.

### 2.8. Data Quality Assurance

To assure the reliability and validity of the test, good-quality and well-defined reagents were used. The patient was instructed on how to collect specimens, and the questionnaire was checked and tested before the actual data collection. Standard operational procedures were used throughout the study period to avoid personal errors; professionally skilled laboratory personnel examined randomly selected slides. The result was carefully recorded, and the data was analyzed appropriately.

### 2.9. Ethical Consideration

The study was carried out after the ethical approval was obtained from the institutional review committee of the College of Health Sciences at Woldia University (reference number: WLDU/AC-CBE/018). A support letter from the Department of Medical Laboratory Sciences was obtained and given to Woldia Comprehensive Specialized Hospital. Written informed consent and assent were obtained from each participant prior to enrollment in the study. Privacy of participants was safeguarded by maintaining strict confidentiality of their personal information to avoid potential harm. Additionally, all laboratory results were kept confidential. Since those were stored in a file using codes without the study participant's name The study participants who were positive for malaria parasites were linked to the hospital and health centers for appropriate treatment and management.

## 3. Results

### 3.1. Sociodemographic Characteristics

Of the 192 study participants, 106 (55.2%) were male and 86 (44.8%) were female. The age group of 16 to 49 years old accounted for the largest proportion (67.2%), while the age group of children under five years old accounted for the lowest proportion (5.2%). In terms of respondents' educational status, the majority were in elementary school (53, 27.6%), followed by illiterates (43, 22.3%), secondary school (42, 21.9%), diploma holders (40, 20.8%), and undereducated people (14, 7.3%) (Table 1).

### 3.2. Malaria Prevalence

Out of the 192 blood samples examined microscopically, 16 tested positive for malaria, resulting in an overall prevalence rate of 8.3%. The study identified two *Plasmodium* species: *P. falciparum*, which was more dominant at 4.7%, and *P. vivax* at 2.6%. Mixed infections of both species accounted for 1.0%. Malaria infection was found across all age groups and in both male and female subjects. The prevalence was higher in males (11.4%) compared to females (5.8%). Malaria infection among age groups has a higher prevalence proportion than the 49-age group (10.3%), followed by the under-five-age group (10%). Malaria infection was observed among rural residents and urban residents at nearly equal rates (8.3%). Concerning educational status, the highest prevalence of the disease was found among individuals who attended secondary school (Grades 9–12) at 11.9%. This was followed by those in primary school at 9.4%, while the lowest prevalence was seen in individuals with diplomas and higher educational levels at 2.5%. Malaria infection is more common among patients who have stagnant water surrounding their residence (13.2%) than those who do not (4.0%), and failing to utilize bed nets has a frequency of 11.4%. In the multivariate analysis, the association with an adjusted odds ratio (AOR) of 2.483, 95% CI of 1.453–11.54, and *p* value of 0.021 was identified as a significant explanatory variable for *Plasmodium* infection (Table 2).

### 3.3. Prevalence and Distribution of ABO Blood Group Types

The most common ABO blood group types were A (59/30.7%), B (49/25.5%), AB (47/24.5%), and O (37/19.3%). According to this finding, the most common ABO blood type was A (59/30.7%), followed by type B (49/25.5%). The magnitude of blood types AB and O was 47 (24.5%) and 37 (19.3%), respectively (Table 3).

### 3.4. Malaria and ABO Blood Group Association

The highest prevalence of malaria (11.9%) was observed in individuals with blood group A, followed by those with blood groups B (8.2%) and AB (6.4%). Individuals with blood group O had the lowest prevalence at 5.4%. The distribution of malaria infection among the ABO blood groups was ranked in descending order as A > B > AB > O. Similarly, the magnitude of the risk of malaria infection in each blood group compared to the “O” type was assessed using COR and AOR at 95% CIs using a logistic regression model. Individuals with the A blood type had a 2.35-fold higher risk of malaria infection (AOR = 2.359, 95% CI: 1.03–12.289, *p* = 0.03) than those with the O blood type. However, in the other blood types, the relationships were not statistically significant (*p* > 0.05) (Table 4).

## 4. Discussion

The study was carried out at Woldia Comprehensive Specialized Hospital. The total frequency of microscopically confirmed malaria parasites was 8.3%. The prevalence rate of malaria parasites detected in this study is lower than 8.5%, 17%, and 50.5%, as reported by Mekaneeyesus Primary Hospital [32]; Metema Hospital, Northwest Ethiopia [35]; and Northwestern Ethiopia [17], respectively. The observed disparities in prevalence rates may be attributed to variations in vector abundance, malaria seasonality, control measures, study population, and sample size. The predominant *Plasmodium* species identified in this study was *P. falciparum* (4.7%), followed by *P. vivax* (2.6%), with mixed infections of both species accounting for 1.0%. This finding aligns with several previous studies conducted in various regions of Ethiopia, which also reported the predominance of *P. falciparum* followed by *P. vivax* and mixed infections in Metema Hospital, Northwest Ethiopia; Northcentral Ethiopia; and North Gondar [35–37]. But this study disagrees with previous studies conducted in South Ethiopia [38] and East Gojjam, Ethiopia [39], where the frequency of *P. vivax* was greater than that of *P. falciparum*. This might be due to variations in geographical location, sample size, and laboratory technique.

Malaria was more common in men (10.4%) than in women (5.8%) in this research. This is consistent with prior research undertaken in Northwest Ethiopia [40, 41]; North Ethiopia [42]; and East Gojjam, Ethiopia [39], where the frequency of malaria was greater in males than in females. This may be attributed to the involvement of males in irrigation and other outdoor activities, which likely increases their exposure to malaria vector bites and the subsequent development of the disease compared to females.

Reports from various studies regarding the potential association between ABO blood groups and malaria risk in different populations are contradictory. The highest prevalence of *Plasmodium* infection (11.9%) was observed among individuals with blood group A, followed by those with blood groups B (8.2%) and AB (6.4%). Individuals with blood group O were the least affected, with a prevalence of 5.4%. This finding aligns with a previous study conducted at Mekane Eyesus Primary Hospital [32]. However, this study was inconsistent with the previous study conducted in Ethiopia, where blood group O was the dominant blood type in uncomplicated malaria (45.7%) [43]. This might be due to variations in genetic factors, environmental conditions, and methodology of the study. The current study identified a 2.35-fold increased risk of *Plasmodium* infection in individuals with blood type A (*p* = 0.030). It was also found that individuals with blood group O were less susceptible to severe malaria compared to those with other blood groups. The variation in susceptibility to *P. falciparum* infection and the severity of the disease may be attributed to differences in the resetting ability of RBCs among the various ABO blood groups, with individuals having blood group O exhibiting a diminished resetting potential [44].

## 5. Conclusion

The overall prevalence of microscopically confirmed malaria parasites was 8.3%. Two species of *Plasmodium* were identified: *P. falciparum*, which was dominant, and *P. vivax*. Additionally, the likelihood of contracting malaria was significantly higher in individuals with blood group A. In contrast, individuals with blood group O were less susceptible to severe malaria compared to those with other blood groups.

## 6. Recommendations

Based on the findings of this study, we recommend that other researchers conduct additional scientific investigations into the association between malaria infection and ABO blood group phenotypes. Furthermore, the relationship between malaria and the ABO blood group should be evaluated taking into account the minor ABO blood group, allelic frequency, and genotypic frequency of the ABO blood type.

## Figures and Tables

**Table 1:** Sociodemographic characteristics of the study participants at Woldia Comprehensive Specialized Hospital (2023).

**Variable**	**Frequency**	**Percent (%)**
Age		
≤ 5	10	5.2
6–15	24	12.5
16–49	129	67.2
> 49	29	15.1
Sex		
Male	106	55.2
Female	86	44.8
Residence		
Rural	96	50.0
Urban	96	50.0
Educational status		
Illiterates	43	22.4
Primary school	53	27.6
Secondary school	42	21.9
Diploma and above	40	20.8
Undereducated	14	7.3

**Table 2:** Prevalence of malaria and association factor at Woldia Comprehensive Specialized Hospital (2023).

**Variable**	**No. of examinations** **N** ** (%)**	**Malaria positive** **N** ** (%)**	**Malaria negative** **N** ** (%)**	**Crude OR (95% CI)**	**p** ** value**	**AOR (95% CI)**	**p** ** value**
Age							
≤ 5	10 (5.2)	1 (10)	9 (90)	0.83 (0.383–1.8)	0.637		
6–15	24 (12.5)	1 (4.2)	23 (95.8)				
16–49	129 (67.2)	11 (8.5)	118 (91.5)				
> 49	29 (15.1)	3 (10.3)	26 (89.7)				
Sex							
Male	106 (55.2)	11 (10.4)	95 (89.6)	1.876 (0.626–5.623)	0.261		
Female	86 (44.8)	5 (5.8)	81 (94.2)				
Education							
Undereducated	14 (7.3)	1 (7.1)	13 (92.9)	1.206 (0.785–1.853)	0.392		
Illiterates	43 (22.4)	4 (9.3)	39 (90.7)				
Primary school	53 (27.6)	5 (9.4)	48 (90.6)				
Secondary school	42 (21.9)	5 (11.9)	37 (88.1)				
Diploma and above	40 (20.8)	1 (2.5)	39 (97.5)				
Residence							
Rural	96 (50.0)	8 (8.3)	88 (91.7)	1 (0.359–2.783)	1		
Urban	96 (50.0)	8 (8.3)	88 (91.7)				
Bed net							
Yes	104 (54.2)	6 (5.8)	98 (94.2)	0.478 (0.166–1.371)	0.012	2.483 (1.453–11.54)	0.021
No	88 (45.8)	10 (11.4)	78 (88.6)				
Stagnant water							
Yes	92 (47.9)	12 (13.2)	78 (86.8)	3.671 (1.145–11.447)	0.029		
No	100 (52.1)	4 (4.0)	97 (95.0)				
Opening house							
Yes	79 (41.1)	4 (5.1)	75 (94.9)	0.449 (0.139–11.447)	0.18	0.418 (0.127–1.381)	0.153
No	113 (58.9)	12 (10.6)	101 (89.4)				

**Table 3:** Prevalence and distribution of ABO blood group systems at Woldia Comprehensive Specialized Hospital (2023).

**ABO blood group types**	**Prevalence in number (percent)**
A	59 (30.7%)
B	49 (25.5%)
AB	47 (24.5%)
O	37 (19.3%).

**Table 4:** Association between the ABO blood group and malaria frequency at Woldia Comprehensive Specialized Hospital (2023).

**Blood group**	**No. of examinations**	**Microscopic examination**				**Total**	**COR (95% CI)**	**p** ** value**	**AOR (95% CI)**	**p** ** value**
		**Pf**	**Pv**	**Mixed**	**Noninfected**					
A	59	4	2	1	52	59	1.35 (0.828–2.216)	0.010	2.359 (1.03–12.289)	0.03
B	49	1	2	1	45	49	1.575 (0.268–9.255	0.615	2.356 (0.45–12.29)	0.31
AB	47	2	1	0	44	47	1.295 (0.201–8.337)	0.786	1.575 (0.268–9.255)	0.62
O	37	2	0	0	35	37	0.254 (2.54–3.45)	0.325	1.295 (0.201–8.337)	0.79
Total	192	9	5	2	17	192				

## Data Availability

We confirm that all data for this manuscript are available. Interested parties can contact Wagaw Abebe to request the data. This manuscript provides a summary of all datasets generated and/or analyzed during the present study.

## Nomenclature

AOR: adjusted odds ratio
CI: confidence interval
COR: crude odd ratio
RBC: red blood cell
SPSS: Statistical Package for the Social Sciences
WHO: World Health Organization
